# Combined Resistance and Plyometric Training Is More Effective Than Plyometric Training Alone for Improving Physical Fitness of Pubertal Soccer Players

**DOI:** 10.3389/fphys.2019.01026

**Published:** 2019-08-07

**Authors:** Firas Zghal, Serge S. Colson, Grégory Blain, David G. Behm, Urs Granacher, Anis Chaouachi

**Affiliations:** ^1^Education, Motor Skills, Sports and Health, Higher Institute of Sport and Physical Education of Sfax, University of Sfax, Sfax, Tunisia; ^2^Université Côte d’Azur, Laboratoire Motriciteì Humaine Expertise Sport Santeì, Nice, France; ^3^School of Human Kinetics and Recreation, Memorial University of Newfoundland, St. John’s, NF, Canada; ^4^Division of Training and Movement Science, University of Potsdam, Potsdam, Germany; ^5^Tunisian Research Laboratory “Sports Performance Optimization”, National Center of Medicine and Science in Sports (CNMSS), Tunis, Tunisia; ^6^AUT University, Sports Performance Research Institute New Zealand, Auckland, New Zealand; ^7^PVF Football Academy, Hu’ng Yên, Vietnam

**Keywords:** strength, power, rate of torque development, jumping, running

## Abstract

The purpose of this study was to compare the effects of combined resistance and plyometric/sprint training with plyometric/sprint training or typical soccer training alone on muscle strength and power, speed, change-of-direction ability in young soccer players. Thirty-one young (14.5 ± 0.52 years; tanner stage 3–4) soccer players were randomly assigned to either a combined- (COMB, *n* = 14), plyometric-training (PLYO, *n* = 9) or an active control group (CONT, *n* = 8). Two training sessions were added to the regular soccer training consisting of one session of light-load high-velocity resistance exercises combined with one session of plyometric/sprint training (COMB), two sessions of plyometric/sprint training (PLYO) or two soccer training sessions (CONT). Training volume was similar between the experimental groups. Before and after 7-weeks of training, peak torque, as well as absolute and relative (normalized to torque; RTD_*r*_) rate of torque development (RTD) during maximal voluntary isometric contraction of the knee extensors (KE) were monitored at time intervals from the onset of contraction to 200 ms. Jump height, sprinting speed at 5, 10, 20-m and change-of-direction ability performances were also assessed. There were no significant between–group baseline differences. Both COMB and PLYO significantly increased their jump height (Δ14.3%; ES = 0.94; Δ12.1%; ES = 0.54, respectively) and RTD at mid to late phases but with greater within effect sizes in COMB in comparison with PLYO. However, significant increases in peak torque (Δ16.9%; *p* < 0.001; ES = 0.58), RTD (Δ44.3%; ES = 0.71), RTD_*r*_ (Δ27.3%; ES = 0.62) and sprint performance at 5-m (Δ-4.7%; *p* < 0.001; ES = 0.73) were found in COMB without any significant pre-to-post change in PLYO and CONT groups. Our results suggest that COMB is more effective than PLYO or CONT for enhancing strength, sprint and jump performances.

## Introduction

Besides soccer-specific technical skills, individual and team tactical knowledge and particularly adequate levels of physical fitness constitute important prerequisites for success in soccer (Stolen et al., 2005). Typical soccer movements such as tackling, jumping, sprinting, shooting, and rapid change-of-directions require high levels of physical fitness (Meylan and Malatesta, 2009). These actions are crucial for optimal performance not only in adult (Faude et al., 2013) but also in youth soccer (Thomas et al., 2009; Marques et al., 2013; Michailidis et al., 2013; Sohnlein et al., 2014), even though they represent only a small but often decisive percentage of total match time. Therefore, identifying effective training methods to optimize performance are vital, especially in young soccer players.

Currently, it is well-established that resistance training (RT) as well as plyometric (PLYO) or plyometric/sprint training are safe and appropriate tools for improving physical fitness of sedentary youth aged 11–12 years (Ingle et al., 2006) or young soccer players aged 10–15 years (Diallo et al., 2001; Kotzamanidis, 2006; Thomas et al., 2009; Michailidis et al., 2013; Sohnlein et al., 2014; de Villarreal et al., 2015). Henceforth, combining RT with PLYO training has become an increasingly popular training method showing better results for proxies of muscular power when compared to PLYO or RT separately (Adams et al., 1992; Fatouros et al., 2000). In soccer, the effectiveness of combined RT with PLYO training was also well established in young (<15 years) soccer players (Franco-Marquez et al., 2015) displaying greater gains in sprint speed, countermovement jump height and squat movement velocity than the typical soccer training alone. However, such improvements have also been demonstrated in 13–15 year old young soccer players with PLYO training combined or not with sprints (Marques et al., 2013; de Villarreal et al., 2015). Unlike RT which requires some training facilities (economic consequences, materials, schedule availability, qualified fitness coaches), PLYO and/or sprint training provide a greater ease of application or performance. Therefore, the usefulness and benefits of supplementing resistance training to PLYO or PLYO/sprint training can be questioned, if this latter already provides the optimal and desired athletic performance improvements in young soccer players aged 13–15 years (Marques et al., 2013; de Villarreal et al., 2015). Previous researchers have directly compared the effects of combined (RT + PLYO training) versus PLYO training but none of them were conducted with young soccer players (Ford et al., 1983; Bauer et al., 1990; Adams et al., 1992; Lyttle et al., 1996; Fatouros et al., 2000; Arabatzi et al., 2010; de Villarreal et al., 2011).

The above reported studies were associated with several limitations and conflicting results. While some studies have found greater improvements with combined training (Adams et al., 1992; Fatouros et al., 2000), others were equally effective with both training methods (Ford et al., 1983; Bauer et al., 1990; Lyttle et al., 1996; Arabatzi et al., 2010; McKinlay et al., 2018). These discrepancies might have several explanations including, among others, the nature and/or type of the training protocol, the training stimulus, or the assessed population. A major limitation of the above-cited studies was that authors did not equate total workload between groups. It is also important to note that all aforementioned studies were performed with sedentary men (Adams et al., 1992; Fatouros et al., 2000) and not young (soccer) athletes. Of note, the initial training status of participants as well as growth and maturation are relevant determinants of adaptations related to RT or PLYO training (Adams et al., 1992; de Villarreal et al., 2011; Lehnert et al., 2013). It is therefore possible that elite power sport athletes such as soccer players would not display the same magnitude of improvement or need different training stimuli with combined or PLYO training (Lehnert et al., 2013). For instance, short-term (6–7 weeks) PLYO training has been demonstrated as an insufficient stimulus to induce further adaptations in well-trained players (Slimani et al., 2016). Nevertheless, supplementing low-load high-velocity RT with PLYO training could, perhaps, overcome this constraint by inducing physical fitness and performance improvements despite the shortening of the training period (Ford et al., 1983). If such an assumption is corroborated, combined-training (COMB) could provide a less stressful alternative program than PLYO/sprint training alone reducing injury risks that could be associated with a singular focus on plyometric exercises (Wang and Zhang, 2016).

Accordingly, the purpose of the present study was to examine the effects of a 7 weeks COMB compared to PLYO/sprint (PLYO) or an active control (CONT) on selected components of physical fitness in young soccer players. With reference to the relevant literature (Adams et al., 1992; Fatouros et al., 2000; de Villarreal et al., 2011), we hypothesized that COMB would result in greater gains in the above mentioned measures compared to single-mode PLYO/sprint or CONT.

## Materials and Methods

### Experimental Approach to the Problem

**FIGURE 1:**
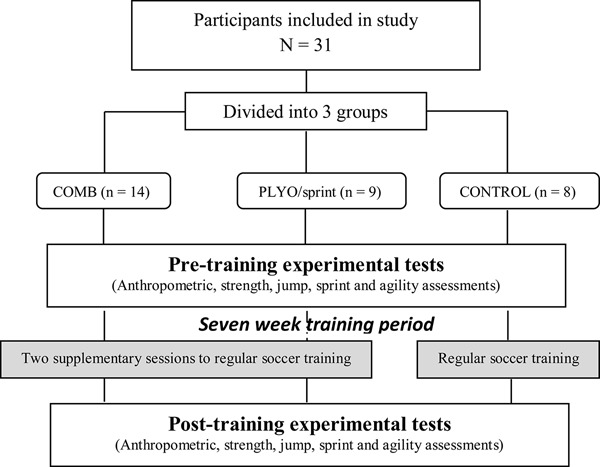
Experimental design. COMB: combination of 1 plyometric/sprint and 1 traditional resistance training sessions per week. Plyo/sprint: addition of 2 plyometric and sprint training sessions per week.

To investigate the research question, we matched pubertal soccer players and subsequently randomly divided them into two training groups and an active CONT: (1) combination (COMB), (2) PLYO/sprint, and (3) CONT groups. This design enabled us to examine whether or not the addition of higher velocity RT to PLYO/sprint training (COMB group) improved physical performances-related to skills in young soccer players to a greater extent than PLYO/sprint training alone. The outcome variables were jump performances (squat jump [SJ], countermovement jump [CMJ] and drop jump [DJ] heights), knee extensor isometric mechanical parameters (peak torque, RTD and RTD_*r*_), sprint (5, 10, 20 m) and change-of-direction ability (505 tests) performances (Figure 1).

### Participants

An *a priori* power analysis was computed with an assumed Type I error of 0.01 and a type II error rate of 0.10 (90% statistical power) to detect significant and medium-sized group by time interactions for countermovement jump height according to the study of Rodriguez-Rosell et al. (2017). Considering a potential drop-out rate of 20%, 37 young male soccer players belonging to the same club level soccer team (A and B level squads) were enrolled in this study. Six players were excluded from the study because they were injured or were absent from the post-testing session. As a result, the training program was completed by thirty-one players. All participants had soccer trained for at least 4 years, no strength training experience, injury free for at least 6 months before participation in this study. The pubertal soccer players were divided by randomization into two training groups and an active CONT: (1) combination (COMB; *n* = 14), (2) PLYO/sprint; (*n* = 9), and (3) CONT; (*n* = 8) groups, using a simple computer generated random number list. No significant between-group baseline or pre-to-post-training differences in anthropometrics were observed. except the thigh circumference that increased significantly post-training in the COMB group only (0.47 ± 0.04 to 0.49 ± 0.04 m; *F*_(2,28)_ = 18.19; *p* < 0.001; η^2^ = 0.56) (Table 1).

**TABLE 1:** Anthropometric characteristics of combined (COMB), plyometric (PLYO), and control (CONT) groups before and after training.

**Group**	**COMB (*n* = 14)**		**PLYO (*n* = 9)**		**CONT (*n* = 8)**	
	**Pre**	**Post**	**Pre**	**Post**	**Pre**	**Post**
Age (years)	14.5±0.5	14.5±0.5	14.5±0.5	14.6±0.5	14.6±0.5	14.7±0.5
Body height (m)	1.72±0.06	1.73±0.05	1.71±0.07	1.72±0.06	1.71±0.07	1.72±0.4
Body mass (kg)	60.2±6.5	62.3±7.2	59.3±6.5	60.2±7.7	61.3±4.3	60.2±6.7
Tanner stage	3.57±0.14	3.64±0.13	3.44±0.18	3.44±0.18	3.50±0.19	3.63±0.18
Thigh circumference (m)	0.47±0.04	0.49±0.04^*⁣**^	0.47±0.04	0.48±0.04	0.48±0.03	0.48±0.03
Training experience (years)	5.71±0.40	5.71±0.40	5.11±0.42	5.11±0.42	5.63±0.32	5.63±0.32

*^∗∗∗^Significant differences between pre-training and post-training values (*p* < 0.001).*

All participants were in the pubertal stage and this was determined by the development of pubic hair, according to the tanner method (Beunen et al., 1992) at the beginning and the end of the 7-week training period by the same investigator (medical doctor). Players who were not classified as tanner stage 3–4 for pubic hair growth and genital development were excluded. The players and their parents/legal guardians were fully informed of the procedure and the risks involved and gave their written informed consent. The experimental protocol was fully approved by the Ethics Committee of the National Center of Medicine and Science of Sports of Tunis (CNMSS) before the commencement of the assessment.

### Testing Procedures

Before the study and prior to setting up of tests, all players completed a 2-week orientation period (two sessions/week). Before the beginning of tests, several warm-up sets including a 5-min jog at a self-selected comfortable pace followed by a 5-min series of dynamic stretching and accelerations were performed. To examine the training effects, the tests were completed before and after seven weeks of the training program over two consecutive days in the following fixed order: anthropometric assessment, maximal voluntary isometric contraction (MVIC) and jump measurements on the first day and then tests for sprint and change-of-direction ability on the second day. In addition, a 2-min recovery was allowed between all repetitions of each test to limit the effects of fatigue. The participants were asked to wear the same clothing and footwear and to avoid strenuous activity during the 24-h proceeding each test session. In addition, post-testing was carried out three to 5 days after the last training session to ensure optimal recovery from the acute effects of training (Weiss et al., 2003), using the same timeline and procedures as during pre-testing. Testing was completed at the same time on each testing day by the same trained investigators.

### Training Program

The training period lasted 7-weeks and was conducted during the competitive season (Table 2). All players followed an in-season soccer training program four times a week plus competition with each session lasting ∼90 min focused on technical and tactical skills development. Two sessions a week of combined (one low-load high-velocity RT and one PLYO/sprint training), two PLYO/sprint sessions or two soccer training sessions were added to the regular soccer program for COMB, PLYO/sprint and CONT groups, respectively. These two supplementary sessions were organized as follows: one (RT or PLYO/sprint for COMB or PLYO/sprint groups, respectively) training session carried out at the beginning (48 h after the last game) and a second (PLYO/sprint training for both groups) in the middle of the week (72 h before the next game). This procedure ensured that players were almost totally recovered before the first training session and they were not fatigued for the next competition. The RT loads used by each player were assigned according to 1-RM. The intensity and the volume were progressively increased respectively from 30 to 60% 1-RM and from 2 sets of 6 repetitions to 4 sets of 8 repetitions (separated by 3-min rests) over the 7-week training period. During each repetition, all players were instructed to lift the load as fast as possible in the concentric phase and to gradually resist in the eccentric phase. Exercises during high-velocity RT included knee extensions, leg press and the back half squat using barbells. The PLYO/sprint exercises were introduced one (COMB) or two times a week (PLYO/sprint) into a soccer training session. PLYO/sprint exercises were performed on a soccer/football pitch. Each repetition of PLYO work was followed by sprints at various distances ranging from 5 to 20 m, which was progressively increased over the 7-week training period. Progressive overload principles were also incorporated into the PLYO program by increasing the hurdle-jump height (from 30 to 60 cm) and the number of foot contacts and by varying the complexity of the proposed exercises in PLYO/sprint sessions. Each RT or PLYO/sprint training session lasted ∼40 min and was preceded by the same 10 min dynamic warm up. All training sessions were supervised by both researchers and the team’s training coach in order to ensure optimal execution and record the compliance and individual workout data during each training session. To ensure equal training load between experimental groups, the volume (training weeks, sets, repetitions and duration) and the intensity (from 30 to 60% of the 1-RM or from 30 to 60-cm height for COMB or PLYO/sprint groups, respectively) of work were matched between RT and PLYO training sessions at the beginning of the week. Moreover, the PLYO/sprint training sessions performed in the middle of the week for both COMB and PLYO/sprint groups was similar in terms of volume and intensity. Since the CONT players were from the same soccer team with the same coaches as the experimental participants, they all conducted approximately the same volume of regular soccer training.

**TABLE 2:** Resistance and plyometric training program of experimental groups.

**Weeks**		**W1**		**W2**		**W3**		**W4**		**W5**		**W6**		**W7**	
**Sessions**		**1**	**2**	**3**	**4**	**5**	**6**	**7**	**8**	**9**	**10**	**11**	**12**	**13**	**14**
**Resistance training**	*Leg extension*	2 × 8		3 × 6		2 × 8		3 × 8 (4 at		4 × 8 (4 at		3 × 8		4 × 6	
	*Leg press*	40% 1RM		50% 1RM		60% 1RM		60% + 4 at 30% 1RM)		60% + 4 at 30% 1RM)		50% 1RM		60% 1RM	
	*Half squat*														
**Plyometric training**	*Vertical hurdle jumps/sprint*		2 × 8 (40 cm)		3 × 6 (50 cm)		2 × 8 (60 cm)		3 × 8 (60–30 cm)		4 × 8 (60–30 cm)		3 × 8 (50 cm)		4 × 6 (60 cm)
	*Lateral hurdle jumps/sprint*		2 × 8 (40 cm)		3 × 6 (40 cm)		2 × 8 (60 cm)		3 × 8 (60–30 cm)		4 × 8 (60–30 cm)		3 × 8 (50 cm)		4 × 6 (60 cm)
	*Stride hurdle jumps/sprint*		2 × 8 (40 cm)		3 × 6 (40 cm)		2 × 8 (60 cm)		3 × 8 (60–30 cm)		4 × 8 (60–30 cm)		3 × 8 (50 cm)		4 × 6 (60 cm)

Total repetitions (three exercises)		48	48	54	54	48	48	72	72	96	96	72	72	72	72

*w = weeks.*

Cross contamination of the two groups was not a major concern as differences in training contents between the experimental groups were not substantial. The training interventions were structured so that players from one group or the other were not under the impression that they did not receive an intervention that may have been helpful to promote their physical fitness. In addition, the same coaches were responsible for training of the two groups further enhancing the impression that both groups were receiving a similar intervention. Furthermore, training volumes were similar between groups and the observers/examiners were blinded to group allocation.

Moreover, before and throughout the experiment, an assessment of the internal training load including volume and intensity of each training session and weekly training load was monitored on the basis of the rating of perceived exertion (RPE) scale according to the procedures proposed by Impellizzeri et al. (2004) and previously applied in soccer (CR10-scale). This RPE method was expressed in arbitrary units (AU) and computed by multiplying the training duration (in minutes) by the session RPE score to obtain a daily training load. The weekly training load was thereafter determined by summing the daily training loads for each player during each week. About 30-min after each training session, players were asked to rate the global intensity of the entire workout session using the category ratio-10 RPE scale.

### Assessment of Anthropometrics, Knee Extensors Maximal Voluntary Isometric Contraction and Rate of Torque Development

Thigh muscle volume was estimated from pre-to-post-training by multiple measurements of skinfolds, circumferences, and diameters of the lower limbs, as detailed elsewhere (Jones and Pearson, 1969). For knee extensor (KE) MVIC participants were seated on an isometric dynamometer (Good Strength, Metitur, Finland) equipped with a cuff (∼2 cm above the lateral malleolus) attached to a strain gauge. Participants stabilized themselves by grasping handles on the side of the chair. Safety belts were strapped across the chest, thighs and hips to avoid displacements. All measurements were taken from the individual’s dominant leg, with the hip and knee angles set at 90° and 70° from full extension, respectively. Each testing session was preceded by a warm-up consisting of several submaximal contractions of the KE muscles at a freely chosen intensity. Thereafter, three, 3 s MVIC trials were performed with rest periods of 2 min between trials. Participants were instructed to extend their knee “as strong and as fast as possible” (Andersen et al., 2010). Online visual feedback of the KE force was provided. The dynamometer force signal was digitized using an acquisition card (Powerlab 16SP, ADInstruments, Australia) and Labchart 7.0 software (ADInstruments, Australia). The sampling frequency was fixed at 2 kHz. The force signal was subsequently multiplied by the lever arm length to obtain knee joint torque. The torque signal was corrected for the effect of gravity on the lower leg (Aagaard et al., 2002). MVIC was therefore defined as the highest peak torque value of the three maximal attempts. The intraclass correlation coefficient (ICC) was 0.84. Rate of torque development (RTD) (Nm s^–1^) was defined as the slope of the torque–time curve (i.e., ΔTorque/ΔTime) from 0–200 ms from the onset of torque (Andersen and Aagaard, 2006). Onset of torque was defined as the instant when the knee extensor torque exceeded the baseline by 2.5% of the baseline-to-peak amplitude (Andersen and Aagaard, 2006). The highest value of the three maximal attempts was used for further analysis with a calculated reliability ICC of 0.82. Normalized RTD (RTD_*r*_) was determined as the slope of the moment-time curve when normalized relative to torque (expressed as % torque s^–1^ × 100) (Aagaard et al., 2002).

### Vertical Jump Performance

Vertical jump performance was measured with three different jump types: (i) SJ, initiated from a knee flexion of 90°, (ii) CMJ and, (iii) DJ. During the performance of the jumps, hands were placed akimbo. The jumps’ flight time and height were calculated using an opto-electric system (Optojump Next, Microgate, Bolzano, Italy: accuracy is within 0.001 s). For each type of jump, 2–3 familiarization trials were practiced before performing three test trials interspersed by a 2 min rest. The best (highest) trial was used for further analysis. The ICCs were 0.90, 0.96, and 0.97 for SJ, CMJ, and DJ, respectively.

### Sprint Time

Participants started the test in a standing position with their anterior foot placed immediately behind the starting line. They initiated the start when they were ready and sprinted over 20-m distance. Sprint time was measured using four electronic photo cells connected to a timer (Brower Timing System, Salt Lake City, 174 UT, United States; accuracy of 0.01 s). A photocell was placed at the start, at 5, 10, and 20-m. The first photocell was positioned at a height of 50 cm from the ground and the photocells of 5, 10, and 20-m were placed at the height of the head of the athletes, in an attempt to standardize the part of the body breaking the photocell. After 1 practice trial, 2 sprint tests were performed, separated by a 5-min recovery period. The fastest trial was used for subsequent analysis. All performance times were recorded with an accuracy of 0.001 s, and the time from 0 to 5-m (ICC = 0.90), 0 to 10-m (ICC = 0.89) and total sprint time from 0 to 20-m (ICC = 0.92) were recorded. These sprint distances were selected because they are the most common during soccer games.

### Change-of-Direction Ability Test

Change-of-direction ability was evaluated with the 505 Test. Marker lines were placed on the ground at 5 and 10-m. The participants accelerated for 10-m in a straight line, there they touched a line with the right or left foot, they turned around, and ran back through the 5-m marker. The time was recorded from the incident when the players first ran through the 5-m marker and it was stopped as they returned through this marker. The time taken from the starting line to 5 and 10-m was measured using photoelectric cells (Brower Timing System, Salt Lake City, 174 UT, United States; accuracy of 0.01 s) and considered for analysis. Each individual performed three trials (ICC = 0.95), and the best one was used for further analysis.

### Statistics

Data are presented as means and standard deviations (SD) in the text and tables. In figures, data are displayed as means and standard errors (SE). The reliability was assessed using the ICC. After normal distribution of data was examined using the Shapiro–Wilk Test, an independent samples *t*-test was calculated to determine significant between group differences in baseline values. To assess and compare the effects of COMB, PLYO/sprint and CONT on variables of physical fitness, a three (group: COMB, PLYO/sprint, CON) × 2 (time: pre-training, post-training) analysis of variance (ANOVA) with time as a repeated within-subject factor was used. If a statistically significant group × time interaction effect was detected, possible significant pre-post-test changes between specific groups were computed using the Newman–Keuls *post hoc* test. For each test, partial eta^2^ values (η^2^) were used for effect size calculation (with ∼0.01 = small effect, ∼0.06 = moderate effect, ≥0.14 = large effect). Additionally, within-group effect sizes (ES) pre-to-post performance changes were calculated using Cohen’s *d* and corrected by Hedge’s g as our sample size was small (<20) to avoid a biased estimation of the population effect size provided by Cohen’s *d*. According to Cohen, ES can be classified as small (0.00 ≤ *d* ≤ 0.49), medium (0.50 ≤ *d* ≤ 0.79), and large (*d* ≥ 0.80). Pearson’s correlation coefficients were calculated to establish the respective relationships between the changes of all measured variables. All data analyses were performed using Statistica (version 8.0, StatSoft, Inc., Tulsa, OK, United States).

## Results

All participants attended all training sessions with no test- or training-related injuries. Results for all measurements are presented in Table 3.

**TABLE 3:** Pre- and post-test values of the measured variables.

**Variable**	**Condition**	**COMB (*n* = 14)**	**PLYO (*n* = 9)**	**CONT (*n* = 8)**	**η^2^ (group) / *p*-value**	**η^2^ (time) / *p*-value**	**η^2^ (time** × **Group) /*p*-value**
**Torque (Nm)**	Pre	296.6 ± 74	293.2 ± 42.8	271.2 ± 36.9	0.13	0.47	0.68
	Post	346.5 ± 78.6^***$££^	293.3 ± 41.4	271 ± 35.3	/	/	/
	Effect size (Cohen’s *d*)	0.58	0.01	0.00	0.15	0.001	0.001
**RTD (Nm s^–1^)**	Pre	278.6 ± 76.0	243.1 ± 62.5	241.0 ± 63.1	0.17	0.46	0.28
0–200 ms	Post	330.7 ± 59.3^***££^	298.5 ± 66.2^∗∗∗^	241.1 ± 64.5	/	/	/
	Effect size (Cohen’s *d*)	0.68	0.77	0.01	0.09	0.001	0.01
**RTD_*r*_ (%Nm s^–1^ × 100)** 0–200 ms	Pre	94.4 ± 16.1	83.2 ± 19.3	89.0 ± 20.9	0.03	0.22	0.27
	Post	96.6 ± 7.6	102.7 ± 20.4^∗∗∗^	89.4 ± 23.2	/	/	/
	Effect size (Cohen’s *d*)	0.16	0.88	0.02	0.67	0.01	0.01
**Jump performance**	Pre	29.5 ± 3.8	29.5 ± 5.4	28.7 ± 1.4	0.14	0.48	0.35
SJ height (cm)	Post	34.6 ± 4.1^***££^	32.8 ± 5.5^∗∗^	28.6 ± 1.5	/	/	/
	Effect size (Cohen’s *d*)	1.15	0.55	0.05	0.13	0.001	0.01
CMJ height (cm)	Pre	35.2 ± 4.0	35.2 ± 6.4	36.4 ± 2.3	0.02	0.55	0.49
	Post	39.5 ± 4.6^***£^	38.3 ± 6.1^∗∗∗^	35.9 ± 2.2	/	/	/
	Effect size (Cohen’s *d*)	0.88	0.44	0.20	0.83	0.001	0.001
DJ height (cm)	Pre	27.5 ± 4.4	24.8 ± 5.9	26.8 ± 4.5	0.10	0.32	0.21
	Post	31.1 ± 3.8^***£^	28.8 ± 5.5^∗∗^	26.6 ± 3.7	/	/	/
	Effect size (Cohen’s *d*)	0.79	0.62	0.06	0.25	0.001	0.04
**Sprint performance**	Pre	1.06 ± 0.07	1.08 ± 0.05	1.06 ± 0.06	0.12	0.14	0.48
5 m-time (s)	Post	1.01 ± 0.06^***$$££^	1.09 ± 0.06	1.06 ± 0.05	/	/	/
	Effect size (Cohen’s *d*)	0.73	0.17	0.06	0.14	0.04	0.001
10 m-time (s)	Pre	1.84 ± 0.08	1.85 ± 0.08	1.88 ± 0.05	0.06	0.00	0.04
	Post	1.83 ± 0.09	1.84 ± 0.09	1.89 ± 0.05	/	/	/
	Effect size (Cohen’s *d*)	0.13	0.14	0.24	0.42	0.94	0.61
20 m-time (s)	Pre	3.16 ± 0.11	3.23 ± 0.19	3.19 ± 0.06	0.09	0.02	0.16
	Post	3.13 ± 0.12	3.22 ± 0.17	3.20 ± 0.08	/	/	/
	Effect size (Cohen’s *d*)	0.26	0.09	0.16	0.27	0.49	0.09
**Agility performance** 10 m-time (s)	Pre	2.50 ± 0.13	2.45 ± 0.16	2.50 ± 0.14	0.02	0.01	0.06
	Post	2.46 ± 0.08	2.47 ± 0.16	2.50 ± 0.13	/	/	/
	Effect size (Cohen’s *d*)	0.31	0.12	0.03	0.82	0.82	0.43

Values are means and standard deviations (SDs). RTD = rate of torque development (i.e., ΔTorque/ΔTime) in 0–200 ms interval from the onset of torque; RTDr = normalized rate of torque development (i.e., slope of the moment-time curve when normalized relative to torque [expressed as % torque s^–1^ × 100]). Significant differences within group: ^*^p < 0.05; ^∗∗^*p* < 0.01; ^∗∗∗^*p* < 0.001. Significant differences between COMB and PLYO groups: ^$^*p* < 0.05; ^$$^*p* < 0.01. Significant differences between COMB and CONT groups: ^£^*p* < 0.05; ^££^*p* < 0.01.

### Peak Torque and Rate of Torque Development (RTD)

The analysis revealed a significant group × time interaction for peak torque [*F*_(2,28)_ = 28.95; *p* < 0.001; η^2^ = 0.68]. *Post hoc* analyses revealed significant increases in peak torque from pre-to-post training in the COMB group (Δ16.9%, *p* < 0.001) without any changes in PLYO/sprint or CONT groups. Greater intra-group ES were found for the COMB (ES = 0.59) when compared to either PLYO/sprint or CONT groups (ES = 0.01 and 0.00, respectively).

Moreover, a significant group × time interaction was observed for both RTD and relative RTD (RTD_*r*_). There was an improved post-training RTD for 0–200 ms in the COMB (*p* < 0.001) and PLYO (*p* < 0.001) groups. Moreover, a significant RTD_*r*_ increase from 0–200 ms in the PLYO/sprint (*p* < 0.001) group (Table 2).

### Sprint Times

Nevertheless, a significant group × time interaction was observed in sprint time at 5-m [*F*_(2,28)_ = 12.68; *p* < 0.001; η^2^ = 0.48]. *Post hoc* test revealed a significant 4.7% faster 5-m sprint time only in the COMB group (from 1.06 ± 0.07 to 1.01 ± 0.06 sec; *p* < 0.001; ES = 0.73) without any significant change for the two others groups.

### Vertical Jump Performances

A group × time interaction was found in SJ [*F*_(2,28)_ = 7.57; *p* < 0.01; η^2^ = 0.35], CMJ [*F*_(2,28)_ = 13.24; *p* < 0.001; η^2^ = 0.49], and DJ height (*F* = 3.58; *p* < 0.05; η^2^ = 0.20), respectively. Further *post hoc* tests showed a significant increase in SJ, CMJ and DJ height performances in both the COMB and PLYO/sprint groups but with a greater within-group ES in COMB (1.15, 0.88, 0.79 for respectively SJ, CMJ, and DJ) when compared to PLYO group (0.55, 0.44, 0.62 for respectively SJ, CMJ, and DJ). Nevertheless, no significant changes were evident in the CONT group between pre- and post-training tests.

### Change-of-Direction Ability

There were no significant interactions observed with change-of-direction ability.

### Correlations

When the data from both groups were pooled, significant positive correlations were observed between the individual relative changes in torque and the individual relative changes in SJ (*r* = 0.36; *p* < 0.05) and CMJ (*r* = 0.41; *p* < 0.05) and sprint (*r* = −0.57; *p* < 0.01). The individual relative changes in SJ also showed a significant positive correlation with the individual relative changes in CMJ (*r* = 0.69; *p* < 0.001) and DJ (*r* = 0.52; *p* < 0.01). However, no significant correlations were found between the individual relative changes in all jump parameters and the individual relative changes in any sprint performances (Table 4).

**TABLE 4:** Relationship between the individual relative changes of the different performance variables.

**Variable**	**Torque**	**SJ**	**CMJ**	**DJ**	**5 m**	**10 m**	**20 m**
**Torque**	1	0.36^*^	0.41^*^	0.08	–0.57^∗∗^	–0.21	–0.22
**RTD_*r*_**							
0–200 ms	0.24	0.25	0.04	0.11	–0.24	–0.03	–0.16
**Jump height**							
SJ	0.36^*^	1	0.69^∗∗∗^	0.52^∗∗^	–0.20	–0.05	–0.04
CMJ	0.41^*^	0.69^∗∗∗^	1	0.48^∗∗^	–0.20	–0.20	–0.20
DJ height	0.08	0.52^∗∗^	0.48^∗∗^	1	–0.10	–0.05	–0.03
**Sprint performance**							
5 m-time	–0.57^∗∗^	–0.20	–0.20	–0.20	1	0.33^*^	0.27
10 m-time	–0.21	–0.05	–0.20	–0.05	0.33^*^	1	0.58^∗∗^
20 m-time	–0.22	–0.04	–0.20	–0.03	0.27	0.58^∗∗^	1

Significant relationship: ^*^p < 0.05; ^∗∗^*p* < 0.01; ^∗∗∗^*p* < 0.001.

## Discussion

The main finding of the present study was the greater effectiveness of combined RT/PLYO/sprint training in comparison with only PLYO/sprint or conventional soccer training for improving strength, sprint and jump measures in pubertal male soccer players.

### Maximal Strength Performance

Our results demonstrated unchanged maximal strength capacities after PLYO/sprint group training Among the studies that have examined the influence of PLYO training on pre-adolescents aged from 5 to 14 years (Diallo et al., 2001; Ingle et al., 2006; Kotzamanidis, 2006; Faigenbaum et al., 2009; Meylan and Malatesta, 2009; Sohnlein et al., 2014), only two studies have measured strength outcomes (Ingle et al., 2006; Faigenbaum et al., 2009) and have shown significant improvements. Our findings were not in accord with these results. However, the first study (Faigenbaum et al., 2009) recruited sedentary participants and thus there could be a greater likelihood of a non-training specific or generalized response to physical activity. As for the second study (Ingle et al., 2006), the authors introduced some resistive exercises to the PLYO training, which could have contributed to the strength improvement. Contrary to PLYO/sprint group in the present study, COMB group demonstrated increased maximal KE strength capacity. The greater effectiveness of COMB vs. PLYO/sprint training in force enhancement has been previously reported (de Villarreal et al., 2011; Faude et al., 2013). However, our study is the first to compare these two different training methods on both maximum force production and RTD of the KE in pubertal soccer players. Even so, these two mechanical parameters are widely thought to be strongly related to explosive movement performances (Tillin et al., 2010), and we also demonstrated positive relationships between KE torque and both sprint (*r* = −0.57) and jump performances (*r* = 0.36 and 0.41 for SJ and CMJ, respectively).

### Jump Height Performance

Contrary to strength results, we demonstrated enhanced jump height performances as a result of PLYO/sprint and COMB training. The magnitude of these improvements (>7%) concurs with previous studies (Meylan and Malatesta, 2009; de Villarreal et al., 2015; McKinlay et al., 2018). The effectiveness of combined RT and PLYO/sprint training on jump performances has been widely reported (Ford et al., 1983; Adams et al., 1992; Fatouros et al., 2000; Arabatzi et al., 2010; de Villarreal et al., 2011), and specifically in young soccer players (Franco-Marquez et al., 2015; Rodriguez-Rosell et al., 2017). Nevertheless, the greater improvements resulting from the COMB compared to PLYO alone were not always evident (Ford et al., 1983; Bauer et al., 1990; Adams et al., 1992; Lyttle et al., 1996; Fatouros et al., 2000; Arabatzi et al., 2010). An interesting result of this study was the absence of significant differences in the magnitude of jump height increases between COMB and PLYO/sprint groups although a greater within-group ES improvement was found in the COMB group in comparison with the PLYO/sprint group. These findings are in agreement with those suggesting a similar extent of jump performance improvements between PLYO and COMB training in physical education students or adults participating in various regional level sports (Ford et al., 1983; Bauer et al., 1990; Lyttle et al., 1996; Arabatzi et al., 2010; de Villarreal et al., 2011). However, the mechanisms for these maximum strength and RTD improvements seem to differ between these different training methods (Arabatzi et al., 2010). Of note, the largest effect size magnitudes were found following COMB for SJ > CMJ > DJ. This indicates that the primary physiological adaptations following COMB did not take place in the SSC, which is illustrated through the lowest ES in DJ performance. Interestingly, single mode PLYO/sprint produced the largest ES for DJ performance which indicates better utilization of the SSC following training.

### Performance Correlations

No significant correlation between jump height performances and mechanical parameters were evident in the present study. This finding was most likely due to the lack of testing specificity when associating isometric measures with dynamic functional athletic performances (Wilson et al., 1995). Such a relationship has been previously reported (Wilson et al., 1995; McLellan et al., 2011). The authors suggested that both maximal strength and RTD are the primary contributors to jumping performances in active participants. McKinlay et al. (2017) found that while absolute isometric peak torque and RTD were not correlated with jump height, there was a significant correlation with relative torque and RTD (normalized to body mass). Moreover, Tillin et al. (2013) suggested that sprint performance was most strongly related to the proportion of maximal force achieved in the initial phase of RTD whereas jump performance was more related to absolute force in the later phase of force-time curve in trained participants. Thus, we displayed unchanged torque and persistent increases in RTD_*r*_ from 0 to 200 ms in the PLYO/sprint group. These results revealed that plyometric/sprint training-related jumping improvements were not due to increased maximal KE torque production. Our findings are in accord with a previous study demonstrating unchanged strength but increased KE RTD up to 100 ms (Kyrolainen et al., 2005), even with high level athletes (Matavulj et al., 2001). The authors concluded that stretch-shortening cycle (SSC) exercises were effective in the strength increase of plantar flexor (PF) but not KE muscles. However, they attributed the jumping performance enhancement to the changes in muscle structure and neural adaptation of PF but also to the modifications in the joint control strategy and/or KE RTD capabilities (McLellan et al., 2011). Moreover, other mechanisms could also be involved in improved RTD and jumping performances such as increased neural activation (Michailidis et al., 2013), tendon stiffness (Burgess et al., 2007), motor coordination (Diallo et al., 2001) and better use of the SSC (Komi and Bosco, 1978). With respect to COMB group, we revealed a greater post-training torque and RTD. Hence, improved jump performances in COMB could be related to both KE maximal strength and RTD improvements. Thus, improved activation of KE ensured by combined RT and PLYO training could be transferred from the knee to the ankle contributing to higher jump performances (Toumi et al., 2004). These findings underpinned the major contribution of KE maximum strength improvement in COMB in enhancing RTD (Aagaard et al., 2002) and therefore jump performances (Tillin et al., 2012) as we reported positive relationships between torque and both SJ (*r* = 0.36; *p* < 0.05) and CMJ (*r* = 0.41; *p* < 0.05) performances. Our high velocity RT may have emphasized neural adaptations in the COMB group. Consequently, combined training seems to be more effective in improving explosive strength qualities than both PLYO/sprint and conventional soccer training influencing the results obtained in sprint performance.

### Sprint Performance

The PLYO/sprint group did not improve their sprint time for any of the distances (5, 10, and 20-m). There is debate regarding the effectiveness of PLYO training for acceleration performance. While some studies demonstrated a decrease (improvement) in sprint time and improved speed capacities in pre-pubertal to adolescents soccer players (Diallo et al., 2001; Kotzamanidis, 2006; Meylan and Malatesta, 2009; de Villarreal et al., 2015), others have not (Ingle et al., 2006; Thomas et al., 2009). These discrepancies might be due to the differences of exercise types (vertical/horizontal displacement in jumping; combined or not by some resistive exercises) employed with PLYO/sprint training. Our results are consistent with those of Thomas et al. (2009) reporting no improvement in any sprint distances from 5 to 20-m in youth soccer players although increased jump performances. The authors suggested that the reduced ground contact times with CMJ and DJ were not short enough to elicit an increased ability to generate explosive ground-reaction forces during sprinting. Our correlation analyses displaying no significant relationship between jump and sprint performances from 5 to 20-m distances confirmed these suggestions. Moreover, the absence of sprint performance improvement following PLYO/sprint training could also be attributed to the lack of specificity in the training (Saez de Villarreal et al., 2013). The incorporation of greater horizontal acceleration (i.e., skipping, jumps with horizontal displacement) would perhaps have reduced the sprinting time (Ramirez-Campillo et al., 2015). Thus, the primarily vertical PLYO exercises could explain the absence of sprint enhancement in the present study. Another explanation relates to the duration of the training intervention. Indeed, Sohnlein et al. (2014) showed that 20 m-sprint performance improved after 16 weeks of plyometric training without any enhancement at fourth, eighth or twelfth weeks. Thus, the 7-weeks PLYO/sprint training period seems to be insufficient to induce any sprinting performance improvement, with well-trained soccer athletes (Potach and Chu, 2000; Lehnert et al., 2013). However, when explosive RT was supplemented with PLYO/sprint training in COMB group, improved speed performance particularly in the initial phase of the sprint was reported. In fact, the acceleration phase in sprint performance is strongly dependent on the reaction time and the athlete’s ability to generate a high RTD and power during propulsion (Slimani et al., 2016). This is consistent with earlier observations for both multiple-joint (Tillin et al., 2013) or isolated KE (Tillin et al., 2010). A previous study has shown that initial explosive-isometric force production is strongly influenced by agonist activation (de Ruiter et al., 2006), although other neural adaptations could also be involved (i.e., increases in motor unit activation and/or firing frequency, synchronization of motor units, decreases in antagonist co-activation) (Folland and Williams, 2007). Our findings confirm previous suggestions of the more beneficial effects of combined RT and PLYO training vs. PLYO training alone on sprint performances (Ford et al., 1983). This study is the first to compare adaptations related to these two training methods on different functional athletic performances related to soccer (i.e., jump, sprint and change-of-direction ability performances) in young soccer players.

### Change of Direction Performance

In respect to change-of-direction ability performance, no group revealed any changes from pre- to post-training. Our findings are in disagreement with previous studies showing improvement of change-of-direction ability assessed with the same test in response to PLYO (Lyttle et al., 1996; Meylan and Malatesta, 2009; Marques et al., 2013; Sohnlein et al., 2014) or combined training (Mujika et al., 2009; Maio Alves et al., 2010) in young soccer players. Other previous studies have also demonstrated unchanged change-of-direction ability despite significant improvements in sprinting (Tricoli et al., 2005; Maio Alves et al., 2010). They rationalized that the performance in movements that demand change-of-direction ability are more dependent on motor control factors than on maximum strength or muscular power. Moreover, as previously mentioned (Maio Alves et al., 2010), the fact that no additional training exercises were implemented in which athletes had to perform changes-in-direction, decelerate, and starts-movements, as demanded in the 505 Test, could explain these results. Hence, it is strongly recommended to introduce sprints with different changes-of-direction immediately after PLYO exercises to better transfer adaptations-related to RT and/or PLYO training into change-of-direction ability performance.

### Limitations

The seven week training program may not have revealed training adaptations that might develop with longer programs and thus could be considered a limitation of the present study. In addition, this study did not include a RT only group as have some previous studies (McKinlay et al., 2018). However, with a finite number of available participants, the inclusion of more groups would have decreased the statistical power of the analysis. Furthermore, as only boys were included as participants, further research should examine whether the present results also apply to female youth and adults. Furthermore, it has been suggested that studies should be restrictive in the number of measures included to minimize chances of type I error inflation. However, post-test statistical power analyses of the study’s measures demonstrated strong statistical power ranging from 0.97 to 0.99, with the exception of the agility measures with a power of 0.61.

## Conclusion

The results of the present study showed that a 7-week in-season combined explosive RT and PLYO/sprint training program was more effective than only PLYO/sprint or conventional soccer training for improving strength (torque), jumping and running performances in pubertal competitive male soccer players. Therefore, soccer coaches could apply this type of training but should combine it with conventional soccer training to optimize its benefits and to transfer them into specific soccer skills in order to increase the explosive performance of their soccer players. Indeed, combined training can be effectively introduced in-season in order to benefit from its positive effects on explosive performances while minimizing overtraining prior to competition.

## Data Availability

The raw data supporting the conclusions of this manuscript will be made available by the authors, without undue reservation, to any qualified researcher.

## Ethics Statement

The experimental protocol was fully approved by the Ethics Committee of the National Center of Medicine and Science of Sports of Tunis (CNMSS).

## Author Contributions

FZ involved in the conceptualization of the study, data assessment, data analysis, and the writing of the manuscript. SC and GB were involved in the conceptualization of the study, data assessment, and the writing of the manuscript. AC, UG, and DB were involved in the conceptualization of the study, data analysis, and the writing of the manuscript.

## Conflict of Interest Statement

The authors declare that the research was conducted in the absence of any commercial or financial relationships that could be construed as a potential conflict of interest.
